# Immune Activation, Cd4+ T Cell Counts, and Viremia Exhibit Oscillatory Patterns over Time in Patients with Highly Resistant HIV Infection

**DOI:** 10.1371/journal.pone.0021190

**Published:** 2011-06-20

**Authors:** Christina M. R. Kitchen, Lilit Yeghiazarian, Rebecca Hoh, Joseph M. McCune, Elizabeth Sinclair, Jeffrey N. Martin, Steven G. Deeks

**Affiliations:** 1 Department of Biostatistics, University of California Los Angeles, Los Angeles, California, United States of America; 2 Environmental Engineering Program, School of Energy, Biological and Medical Engineering, University of Cincinnati, Cincinnati, Ohio, United States of America; 3 HIV/AIDS Program, Department of Medicine, University of California San Francisco, San Francisco, California, United States of America; 4 Division of Experimental Medicine, Department of Medicine, University of California San Francisco, San Francisco, California, United States of America; 5 Department of Epidemiology and Biostatistics, University of California San Francisco, San Francisco, California, United States of America; South Texas Veterans Health Care System, United States of America

## Abstract

The rates of immunologic and clinical progression are lower in patients with drug-resistant HIV compared to wild-type HIV. This difference is not fully explained by viral load. It has been argued that reductions in T cell activation and/or viral fitness might result in preserved target cells and an altered relationship between the level of viremia and the rate of CD4+ T cell loss. We tested this hypothesis over time in a cohort of patients with highly resistant HIV. Fifty-four antiretroviral-treated patients with multi-drug resistant HIV and detectable plasma HIV RNA were followed longitudinally. CD4+ T cell counts and HIV RNA levels were measured every 4 weeks and T cell activation (CD38/HLA-DR) was measured every 16 weeks. We found that the levels of CD4+ T cell activation over time were a strong independent predictor of CD4+ T cell counts while CD8+ T cell activation was more strongly associated with viremia. Using spectral analysis, we found strong evidence for oscillatory (or cyclic) behavior in CD4+ T cell counts, HIV RNA levels, and T cell activation. Each of the cell populations exhibited an oscillatory behavior with similar frequencies. Collectively, these data suggest that there may be a mechanistic link between T cell activation, CD4+ T cell counts, and viremia and lends support for the hypothesis of altered predator-prey dynamics as a possible explanation of the stability of CD4+ T cell counts in the presence of sustained multi-drug resistant viremia.

## Introduction

Current therapeutic strategies for HIV-infected persons include the use of antiretroviral therapy to fully inhibit viral replication, as defined by achieving and maintaining undetectable plasma HIV RNA levels. The vast majority of patients who are treatment naïve and able to adhere to a recommended regimen are able to achieve durable and perhaps indefinite viral suppression. A poorly described but significant subset, however, are not able to achieve this outcome, either due to pre-existing resistance and/or the inability to fully adhere to therapy. Most, but not all, of these patients eventually develop drug resistance mutations and, hence, have limited long-term options for complete viral suppression.

The natural history of incomplete or partial viral suppression with combination therapy is complex. As compared to untreated disease, those who remain on a stable regimen despite the presence of drug-resistance mutations have slower rates of CD4+ T cell decline and a lower risk of progressing to AIDS and/or death [1], [2], [3]. This effect appears to be more strongly associated with failure of protease inhibitor-based regimens than with failure of non-nucleoside reverse transcriptase inhibitor based regimens [1], [4]. Although partial reduction in viral load clearly contributes to the residual benefit of therapy [5], the delayed risk of disease progression in treated versus untreated disease remains significant, even after controlling for viral load [1], [2].

Among untreated individuals, the level of viremia is only partially predictive of the rate of disease progression, as defined by the rate of CD4+ T cell loss and/or by the risk of progressing to AIDS and death [6], [7]. T cell activation (as defined by expression of CD38 and HLA-DR) is an independent predictor of CD4+ T cell loss and disease progression among untreated patients [8], [9]. Theoretically, activated T cells may contribute to a poor prognosis by supporting higher levels of viral replication and/or by causing inflammation-associated damage to the immune system and other organ systems. Given the central role of T cell activation in untreated diseases our previous work explored the impact of drug-resistance on the complex relationship between T cell activation and viral load. We have found that, after controlling for the levels of viremia, CD8+ T cell activation was lower in those with drug resistance than those with wild-type HIV. This effect appeared to be more strongly associated with the presence of protease inhibitor resistance rather than direct exposure to the immunomodulatory effects of protease inhibitors [10].

To understand the role of the T cell activation, progressive immunodeficiency, and drug resistant HIV, we performed detailed immunologic and virologic measurements among a cohort of treated patients with detectable viremia who were maintained on a stable regimen pending more effective therapeutic options. The overall objectives of this prospective cohort were to determine the impact of replicative capacity, T cell activation and HIV-specific T cell response on both viremia and peripheral CD4+ T cell counts over time. In the current analysis, we describe how many of these factors evolve over time. We found that CD4+ T cell activation was most strongly associated with the CD4+ T cell counts while CD8+ T cell activation was more strongly associated with viremia. Spectral analysis [11] in a subset of subjects revealed evidence of oscillatory behavior in CD4+ T cell counts, CD4+ T cell activation, CD8+ T cell activation, and plasma HIV RNA levels. Taken together, the data suggest a mechanistic link between T cell activation, viremia and CD4+ T cell depletion and support the hypothesis of altered predator-prey dynamics as a possible explanation for the stability of CD4+ T cell counts even in the present of sustained multi-drug-resistant viremia.

## Materials and Methods

### Subjects

All subjects were enrolled in the Partial Controllers on Antiretroviral Therapy cohort (PCAT) [12]. All subjects provided written informed consent. The study was approved by the University of California, San Francisco Human Research Protection Program Committee on Human Research, Laurel Heights Panel. Eligibility criteria included having a detectable viral load between 200 and 10,000 copies/mL while on a stable optimized combination antiretroviral regimen. Subjects were enrolled and followed in the period prior to the widespread availability of integrase inhibitors, CCR5 inhibitors, and second generation non-nucleoside reverse transcriptase inhibitors, and had hence had limited options for complete viral suppression. Because of concerns that high level viremia (>10,000 copies RNA/mL) would pose substantial risk to the study participant, subjects were encouraged to consider treatment modification once viral loads increased above this level. We chose a threshold of 10,000 copies of HIV RNA/mL (using the bDNA method) for this study because previous data suggested that viral loads above this threshold might be associated with rapid CD4+ T cell loss. Given this data, we felt that subjects and their health care providers should have no perceived barriers to modifying therapy. For the current analysis, subjects were censored if their HIV RNA levels exceeded this threshold on two subsequent visits or if the optimized antiretroviral therapy was modified or discontinued.

### Immunologic and virologic measurements

Viral load and CD4+ T cell counts were measured every 4 weeks. T cell activation (CD38+/HLA-DR+) was measured every 16 weeks using cytokine flow cytometry, as previously described [12]. HIV replicative capacity was also measured longitudinally, using a modified version of an HIV phenotypic drug susceptibility assay (Monogram Biosciences). Briefly, HIV RNA was extracted from the subject's plasma and the terminal 18 codons of the *gag* gene, the entire *pro* gene, and a portion of the *RT* gene were PCR amplified. The amplified gene segments were inserted into a viral vector containing a luciferase gene. Following a single round of viral replication in the absence of drug, luciferase activity was measured and compared to that for a reference virus (NL4-3).

### Statistical analysis

The primary variables considered for modeling CD4+ T cell counts over time included CD4+ and CD8+ T cell activation, CD4 nadir, duration of infection, HIV-specific T cell response, viral burden, and viral replication capacity. Plotting revealed significant skews in the distributions of CD4+ T cell counts, CD4+ and CD8+ T cell activation, and plasma HIV RNA levels, and these values were log_10_ transformed to meet model assumptions. Given the less frequent measurements of immune activation than CD4+ T cell counts, CD8+ T cell counts, and viral load, we used multiple imputation to address the missingness in the T cell activation and replication capacity data. Missing data can lead to bias in estimates as well as a loss of power [13], [14], [15]. The extent of the bias depends on the cause of the missingness. Multiple imputation can be used when the missing data are “missing at random”, that is the probability of the observation being missing for a particular subject does not depend on the value of the marker, conditional on other observed variables. In this case the assumption was justified in that it was solely a cost issue related to the missingness and not an underlying disease process. Multiple imputation has been used successfully in a wide variety of fields of application from astrophysics [16] to chemistry [17] to clinical research [13], [14], [15], [18]. Multiple imputation has not only been used in regression settings but also used in spectral analysis [16], [17], [19]. Instead of filling in a single value for each missing value, such as last observation carried forward (LOCF), multiple imputation replaces each value with a set of draws from a random sample of plausible values reflecting the uncertainty of the missing values [18]. In this way, one creates multiple datasets, each with different draws from the random sample of the missing values resulting in multiple complete case datasets. Standard statistical techniques can then be applied to each complete case imputed dataset and the results are then combined for the final inference. We used PROC MI in SAS version 9.2 for multiple imputation, using the Markov Chain Monte Carlo (MCMC method) with multiple chains. We discarded the first 8000 iterations as “burn in” and used a total of 10000 iterations for inference. We used the Jeffries (noninformative) prior and took the results of the Expectation-Maximization (EM) algorithm as our starting point. For the multiple imputed estimates, time-series and autocorrelation plots showed that our MCMC sampler was mixing well and that the estimates reached convergence. We then used linear mixed models with random intercepts and slopes to assess the association of T cell activation and viremia in CD4+ T cell counts over time in each imputed dataset. An unstructured covariance matrix was used to assess the serial correlation. This approach allows one to model the association of continuous predictor variables measured longitudinally on a continuous longitudinal outcome variable while accounting for within-subject correlations.

We also measured relative efficiency, which is a function of the number of imputations, *m*, and the percent missingness of the data. It is defined in units of variance as the differential utility of using *m* (in this case m = 30) imputations instead of an infinite number of imputations, where 100% is fully efficient. We conducted 30 imputations because results have shown that although the estimate is unbiased, more imputations are needed than previously suggested to attain good statistical power [20]. SAS Proc MIAnalyze was used for statistical inference of parameters from the 30 imputed complete datasets.

### Spectral analysis

Individual viral and immune cell dynamics were analyzed using spectral analysis [11] in a subset of subjects. Subjects with at least 20 observation points for all compartments (i.e., CD4+ T cell counts, HIV viremia, and T cell activation) were included for analysis (N = 11). Spectral analysis can be used to detect cyclical patterns in data, with the purpose of decomposing complex time series with cyclical components into sinusoidal functions. In a sense, performing spectral analysis on time series data can be compared to putting these data through a prism that identifies the underlying cyclical (sinusoidal) components. As a result, data that might initially look like random noise can be meaningfully interpreted in terms of underlying recurring cycles. The decomposition is performed using the Fourier transform. The time series can then be presented as a mixture of functions expressed as 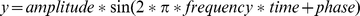. The phase is determined by the initial displacement of the wave at time *t = 0*. In this case the *y* would be the population of interest such as viremia. Amplitude is the maximum height of the wave (in absolute value). Frequency is the reciprocal of the period which is the length of time for the wave to repeat. This signal is thus presented in the frequency domain, where frequency is expressed in units of [1/time] and significant peaks are recorded. Raw data are first de-trended (the mean and the linear fit are subtracted) and then the Lomb-Scargle periodogram is constructed. This algorithm generates a Fourier spectrum for data that are not equally spaced [21]. Signals with peaks below the 50% critical limit were discarded. For this analysis, spectral decomposition was used to determine signal composition for HIV RNA levels, CD4+ T cell activation, CD8+ T cell activation, and CD4+ T cell counts. From the analysis, we determined each signal from noise, identified the presence of oscillatory behavior, and drew conclusions about correlations between the populations. The results show the averages of the quantities of interest average across all imputations.

While spectral analysis can only examine one patient over time, non-linear mixed models can examine all subjects simultaneously. We therefore constructed a non-linear mixed effects model with a random intercept to model log viremia over time. To model the oscillatory trend, we used a mixture of two sine waves with frequencies at 2π and 8π, as suggested by the results of the spectral analysis, to account for any cyclical patterns in the data. We fit a non-linear mixed effects model with random intercept model using the first-order method of Beal and Sheiner [22] that included an indicator for protease inhibitor use, CD8+ T cell activation, baseline viremia, the oscillatory component and an interaction for the oscillatory component and protease inhibitor use. Estimates from the spectral analysis were used as the starting values.

## Results

We examined 54 subjects with partially controlled antiretroviral-resistant viremia on a stable antiretroviral regimen. There were 50 men (93%) and 4 women. The median baseline CD4+ T cell count was 303 cells/mm^3^ (IQR: 210–437) and the median log_10_ plasma HIV RNA level was 3.3 copies/ml (IQR: 2.5–3.7) (Table 1). The baseline percent of activated CD38+HLA-DR+ CD4+ T cells was 6.4% (IQR: 5.0–8.9) and the baseline percent of activated CD38+HLA-DR+ CD8+ T-cells was 21.1% (IQR: 15.1–30.0). The median self-reported CD4 nadir was 82 cells/mm^3^ (IQR: 20–164). Forty (74%) of the subjects were failing on a protease-inhibitor based regimen. Of those, 30 (75%) were failing a boosted protease inhibitor regimen. The median duration of follow-up was 44 weeks (IQR 18.1 to 71.0). Across all subjects, the average rate of CD4+ T cell change was −1 CD4+ T cells/mm^3^/month. Of interest, CD4+ T cell counts increased, on average, by 20 cells/mm^3^ from baseline in subjects taking boosted protease inhibitors, and decreased by 33 cells/mm^3^ in subjects not taking these drugs, although this difference did not reach statistical significance.

**Table 1:** Baseline characteristics of the study group.

Baseline variable	Median	Interquartile range
Age in years	46	42–52
CD4 T+cell, cells/mm3	303	210–437
Plasma HIV RNA log 10 copies/ml	3.3	2.5–3.7
CD4 activation (%)	6.4	5–8.9
CD8 activation (%)	21.1	15.1–30.0
CD4 nadir, cells/mm3	82	20–164
Follow up (weeks)	44	18.1–71.0

	N	Percent
Gender		
Male	50	93
Female	4	7
Protease Inhibitor (PI)		
Boosted	30	55
RTV/LPV	23	43
RTV+other PI	7	13
Non-boosted	10	19
None	14	26
Non-nucleoside RTI	7	13
Nucleoside RTI	53	98

As expected, at baseline there was a negative correlation between log_10_ viral load and CD4+ T cell count (rho = −0.28, p = 0.038). There was also a negative correlation between T cell activation and CD4+ T cell counts with subjects with higher levels of T cell activation having lower CD4+ T cell counts at baseline (rho = −0.38, p = 0.006 for CD4+ T cell activation and rho = −0.30, p = 0.03 for CD8 T cell activation). Log_10_ viremia and both CD4+ and CD8+ T cell activation were strongly correlated (rho = 0.35, p = 0.012 and rho = 0.52, p = 0.0001 respectively). The strong association between log_10_ viremia and CD8 activation persisted across time.

### Longitudinal predictors of CD4+ T cell counts and viremia

We analyzed longitudinal CD4+ T cell counts using a mixed effects model with a random intercept and slope. Correlation over time was modeled using an unstructured covariance matrix. Not surprisingly, CD4+ T cell counts declined over time (adjusted p-value 0.0028). Across all subjects, higher levels of CD4+ T cell counts were temporally associated with lower CD4+ T cell activation, after adjusting for the log plasma HIV RNA levels. For every percentage increase in CD4+ T cell activation, CD4+ T cell counts declined by 2 cells/mm^3^ (adjusted p-value of 0.025; Table 2). Similarly, higher replication capacity was associated with lower CD4+ T cell counts (adjusted p-value 0.041). This effect of CD4+ T cell activation on CD4+ T cell counts was significant in the complete case analysis (data not shown) and remained significant across all multiply imputed datasets. Table 2 gives the estimates from the final mixed effects model as well as the relative efficiencies of the multiple imputation estimates.

**Table 2:** Parameters associated with CD4 T cell count loss among patients with partially-controlled drug resistant viremia using a mixed effects model.

Effect	Estimate	Standard Error	95% CI	P	Relative efficiency
Intercept	437	31.8	(375, 500)	<0.0001	0.996
Time (weeks)	−0.356	0.119	(−0.59, −0.123)	0.0028	0.998
Replication capacity	−0.365	0.174	(−0.715, −0.015)	0.0412	0.975
CD4 activation	−1.695	0.742	(−3.168, −0.222)	0.0246	0.981
Log viral load	−19.95	6.131	(−32.05, −7.85)	0.0014	0.987

The model shows that CD4 T cell count decreased over time. Across all subjects higher levels of CD4 T cell activation were associated with lower levels of CD4 T cell counts controlling for log plasma HIV-1 viremia. Higher replication capacity was also associated with lower CD4 counts.

### Longitudinal predictors of viremia

We analyzed longitudinal plasma HIV RNA levels using a mixed effects model with random intercepts and slopes and an unstructured covariance matrix. Across all subjects, higher levels of plasma HIV RNA levels were temporally associated with lower CD8 T cell activation. For every percentage increase in CD8 T cell activation, log transformed plasma HIV RNA levels increased by 0.02 logs (adjusted P-value<0.0001; Table 3). There was no consistent relationship between replicative capacity and plasma HIV RNA levels. This effect of CD8 T cell activation on plasma HIV RNA levels was significant in the complete case analysis (data not shown) and remained significant across all multiply imputed datasets.

**Table 3:** Parameters associated with log HIV viremia among patients with partially controlled drug resistant viremia using a mixed-effects model.

Effect	Estimate	Standard Error	95% CI	P	Relative efficiency
Intercept	2.81	0.122	(2.57, 3.05)	<0.0001	0.99
Time (weeks)	−0.002	0.0017	(−0.005, 0.001)	0.232	0.997
CD8 activation	0.018	0.0036	(0.010, 0.025)	<0.0001	0.976

The model suggests that increases in CD8 T cell activation was associated with increases in log HIV viremia.

### Immunologic and virologic measures follow an oscillatory pattern

Next, we examined the correlation structure between CD4+ T cells, T cell activation and viral burden over time in eleven subjects using spectral analysis. Spectral analysis allows for modeling the time series of each population as a mixture of sine waves. Each time series was decomposed into a collection of sine waves with different frequencies, amplitude, and phases.

**Figure 1:**
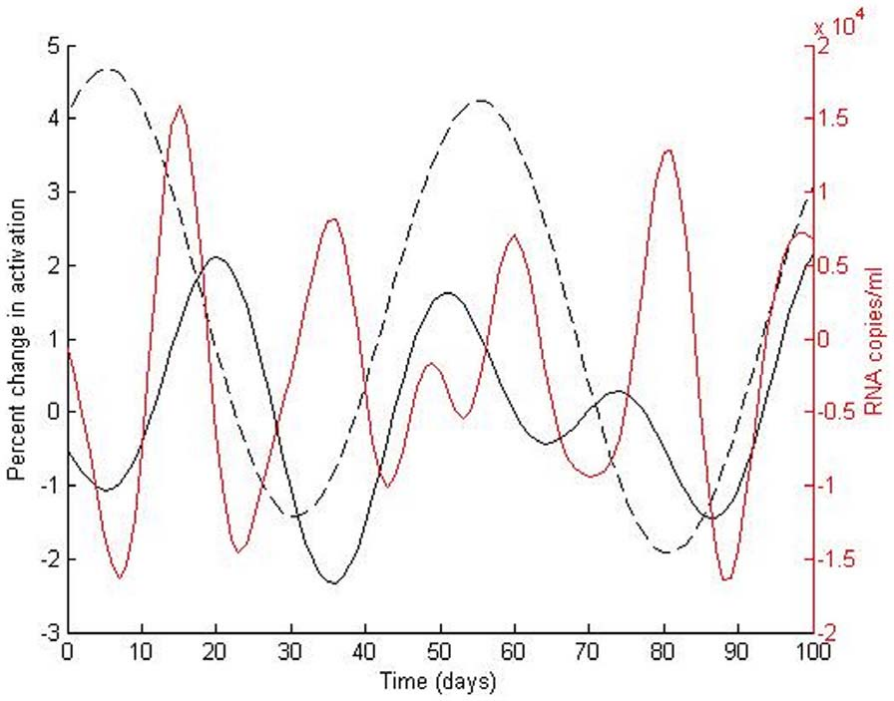
Complex oscillatory behavior in a representative patient. Changes in percent CD4 activation, percent CD8 activation and HIV viremia over time using spectral analysis. Log HIV viremia is in red, CD8 activation is shown with dashed black lines and CD4 activation is shown with the black solid line. Time is in days.

**Figure 2:**
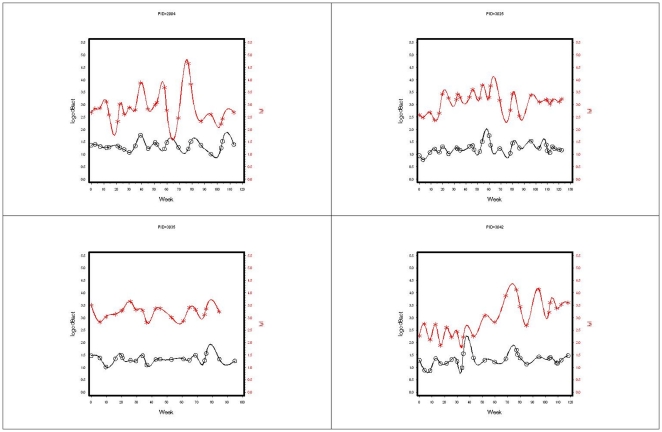
Log viremia and Log CD8 activation over time in 4 patients. Log Viremia (lvl) is shown red and log CD8 activation is in black. Lines were formed using a spline interpolation algorithm.

Table 4 lists the demographic characteristics of the subjects included in this analysis. Subjects in this subset did not differ significantly from subjects not included in this analysis in terms of average level of T cell activation, viremia or CD4+ T cell count. In these eleven subjects we found evidence of oscillatory patterns in the CD4 compartment with subjects exhibiting 2–6 separate waves. Figure 1 shows, for a representative patient, the results from the spectral analysis in terms of T cell activation, log viremia and CD4 counts over time. Using mixed effects models with a compound symmetric correlation structure, we found that subjects with higher levels of CD4+ T cell counts over time had fewer CD4 waves and these waves had lower amplitudes (p = 0.0100 and p = 0.0144 respectively). Similarly, we found cyclical behavior in the T cell activation compartments. Subjects with higher levels of viremia had higher amplitudes of CD8 activation (p<0.0001) and lower phase shifts (p = 0.0001) controlling for CD4 nadir. We found a tight correlation between viremia amplitude and CD8+ T cell activation amplitude (rho = 0.61, p = 0.04). Take together this yields further evidence of the mechanistic linkage between viremia and CD8 T cell activation. Figure 2 plots, for four representative patients, log CD8 activation and log viremia over time.

**Table 4:** Demographic profile of patients included in the spectral analysis.

Patient	Maximum CD4T cell count (cells/mm^3^)	Average CD4T-cell count(cells/mm^3^)	CD4 nadir(cells/mm^3^)	Maximum HIV-1 RNA (copies/mL)	Average HIV-1 RNA (copies/mL)
2004	310	228	2	4.65	2.92
3025	412	273	39	3.78	3.1
3035	319	225	60	3.66	3.24
3037	364	258	220	4.21	3.79
3040	481	365	90	4.27	3.97
3042	232	145	69	4.21	2.97
3077	445	293	285	3.84	3.35
3089	567	376	150	4.11	2.542
3102	420	254	24	4.86	3.95
3135	644	433	250	4.12	2.97
3153	462	356	274	4.78	4.2

Overall, we found strong evidence of complex dynamics, with subjects exhibiting 0 to 7 separate sine waves and with different frequencies for each component. One or greater sine waves is an indication of the presence of oscillatory behavior in activated CD4+ T cells, activated CD8+ T cells, plasma HIV RNA levels, and total CD4+ T cells. Tables 5–8 list, for each subject and each parameter the average of the wave parameters (frequency, amplitude and phase) as well as the total number of waves in the model for CD8 T cell activation, HIV viremia, CD4 activation and CD4 T cell counts respectively. Two subjects did not exhibit oscillatory behavior in viremia. These two subjects also had the lowest CD4 nadirs in the sample with nadirs of 2 and 24 cells/mm^3^). These subjects were also amongst those with the highest maximum viremia. However these subjects did not differ significantly from other subjects in terms of length of follow-up, average CD4+ count or average viremia.

**Table 5:** Spectral analysis estimated parameters for CD8 T cell activation.

	CD8 T cell activation						
Patient	Number of waves	Mean Frequency	Std Dev	Mean Amplitude	Std Dev	Mean Phase	Std Dev
2004	3	0.059	0.039	8.710	1.274	3.163	0.518
3025	3	0.105	0.011	5.838	1.500	2.744	1.999
3035	5	0.067	0.038	5.038	1.833	1.795	1.463
3037	2	0.086	0.077	5.213	3.135	3.738	1.236
3040	4	0.061	0.043	4.543	5.630	2.167	0.658
3042	4	0.018	0.009	42.990	45.323	3.320	2.573
3077	4	0.078	0.062	31.924	21.029	4.064	1.675
3089	3	0.092	0.028	5.354	1.560	2.833	2.128
3102	4	0.073	0.024	16.464	9.540	2.350	1.476
3135	4	0.044	0.024	34.648	32.257	2.643	2.493
3153	3	0.073	0.022	42.089	36.380	2.755	2.333
**Mean**	3.55	0.07	0.03	18.44	14.50	2.87	1.69
**Std Dev**	0.82	0.02	0.02	16.07	16.40	0.67	0.70

For each individual, the mean and standard deviations for wave components for CD8 T cell activation is presented.

**Table 6 pone-0021190-t006:** Spectral analysis estimated parameters for HIV viremia.

	HIV Viremia						
Patient	Number of waves	Mean Frequency	Std Dev	Mean Amplitude	Std Dev	Mean Phase	Std Dev
2004	No oscillations						
3025	4	0.086	0.059	1245.86	692.327	4.436	1.328
3035	4	0.013	0.018	505.27	438.186	1.785	1.175
3037	4	0.034	0.048	3086.03	1102.75	2.734	1.924
3040	4	0.052	0.032	2429.39	704.876	4.759	1.805
3042	2	0.092	0.039	2568.66	483.054	3.000	1.044
3077	5	0.099	0.0319	4333.25	5465.362	4.298	1.3959
3089	2	0.043	0.0521	5882.81	1411.156	5.943	0.1204
3102	No oscillations						
3135	4	0.044	0.0366	8829.99	7456.89	2.727	0.8222
3153	4	0.086	0.0324	41015.96	26361	4.718	2.1039
**Mean**	3.67	0.06	0.04	7766.36	4901.73	3.822	1.30
**Std Dev**	1.00	0.03	0.01	12721.27	8431.66	1.322	0.61

For each individual, the mean and standard deviations for wave components for HIV viremia is presented.

**Table 7 pone-0021190-t007:** Spectral analysis estimated parameters for CD4 T-cell activation.

	CD4 T cell activation						
Patient	Number of waves	Mean Frequency	SD	Mean Amplitude	SD	Mean Phase	SD
2004	6	0.066	0.051	2.438	1.061	2.98	1.37
3025	7	0.059	0.042	4.61	2.475	3.216	1.906
3035	5	0.043	0.034	5.62	2.011	3.191	1.702
3037	2	0.043	0.034	6.18	4.994	3.678	0.965
3040	4	0.069	0.044	8.04	4.561	1.846	1.383
3042	3	0.092	0.027	5.54	1.823	4.219	2.062
3077	5	0.101	0.046	5.42	3.978	3.282	1.932
3089	4	0.053	0.042	3.53	1.48	2.449	2.095
3102	4	0.075	0.04	3.10	3.524	2.339	1.929
3135	4	0.052	0.028	4.27	2.184	2.677	2.826
3153	3	0.090	0.008	3.94	1.434	3.550	0.414
**Mean**	4.27	0.07	0.04	4.79	2.68	3.039	1.69
**Std Dev**	1.42	0.02	0.01	1.59	1.36	0.676	0.64

For each individual, the mean and standard deviations for wave components for CD4 T cell activation is presented.

**Table 8:** Spectral analysis estimated parameters for CD4 T cell count.

	CD4 count						
Patient	Number of waves	Mean Frequency	SD	Mean Amplitude	SD	Mean Phase	SD
2004	2	0.026	0.022	29.93	17.371	3.921	3.328
3025	5	0.088	0.064	173.08	100.792	4.024	2.17
3035	5	0.082	0.039	41.82	25.996	2.702	2.03
3037	5	0.048	0.036	49.70	45.099	2.772	1.071
3040	4	0.045	0.033	94.78	57.969	3.909	1.803
3042	6	0.069	0.04	252.48	220.891	3.281	2.016
3077	3	0.054	0.031	107.63	78.17	2.178	2.56
3089	4	0.093	0.013	70.36	25.979	3.189	1.071
3102	3	0.082	0.01	41.22	17.746	5.017	1.258
3135	4	0.033	0.037	6636.00	7656	2.063	1.755
3153	4	0.081	0.044	157.81	106.193	3.628	1.762
**Mean**	4.09	0.06	0.03	695.89	759.29	3.335	1.89
**Std Dev**	1.14	0.02	0.01	1971.31	2288.15	0.881	0.66

For each individual, the mean and standard deviations for wave components for CD4 T cell counts is presented.

To examine the determinants of oscillatory behavior in these subjects, we constructed a non-linear mixed effects model with random intercepts. We found evidence of an interaction with protease inhibitor use and oscillatory behavior. Subjects who were not on a protease-inhibitor containing regimen had a stronger oscillatory signal than subjects on a protease-inhibitor containing regimen (p = 0.04). In this model CD8+ T cell activation remained a significant predictor of HIV viremia over time (p = 0.0007). Table 9 lists the parameter estimates for this model.

**Table 9:** Parameters associated with log HIV viremia among a subset of patients with partially controlled drug resistant viremia using a non-linear mixed-effects model.

Effect	Estimate	Standard Error	P
Intercept	0.0714	0.4032	0.863
Time (weeks)	0.00614	0.00947	0.1132
CD8 activation	0.8468	0.1739	0.0007
Baseline Viremia	0.6272	0.1171	0.0003
No protease inhibitor	0.2237	0.1128	0.0755
Cyclical component	−0.0183	0.009	0.07
Cyclical component*No PI use	0.02522	0.011	0.0453

The model found evidence of an oscillatory signal in patients whose regimens did not include a protease inhibitor.

## Discussion

T cell activation is a central component of HIV and SIV infection. Among untreated HIV-infected persons, measures of T cell activation predict risk of subsequent disease progression [23], [24]. Among treated subjects, T cell activation is associated with CD4+ T cell count changes during therapy, at least cross-sectionally [10], [25]. T cell activation rather than viral load appears to be the primary characteristic that defines pathogenic versus non-pathogenic SIV infection in non-human primate models [26]. Despite extensive investigation, the temporal associations between immune activation, viral load, and peripheral CD4+ T cell counts have not been fully defined. Here, we examined a unique cohort of HIV-infected persons in which immune activation was thought to have a strong independent effect on disease outcomes, and measured changes in several relevant biologic outcomes over time. We found that, among treated subjects with low levels of detectable drug-resistant viremia (<10,000 copies/mL), higher CD4+ T cell counts were predicted by lower levels of CD4+ T cell activation. In contrast, higher plasma HIV RNA levels were more strongly predicted by higher levels of CD8 T cell activation. We also observed that replication capacity, measured by an *in vitro* assay, was associated with lower CD4+ T cell counts but not with the level of viremia. Using a sophisticated statistical method that seeks to define the temporal cause and effect association of these observations, we found evidence of complex oscillatory behavior in these populations. This behavior was more evident among individuals not receiving a protease-based regimen. This suggests that the various measurements are linked to each other in an as yet poorly defined mechanism, although the temporal changes might be consistent with a previously hypothesized “predator-prey” association, as described below [27].

Our data regarding the relationship between CD4+ T cell activation and CD4+ T cell counts is generally in agreement with prior work in untreated HIV-infected persons. For example, Catalfamo and colleagues reported that the degree of CD4+ T cell activation (or proliferation) is driven by homeostatic responses and that CD8+ T cell activation is mainly a direct pro-inflammatory consequence of viral replication [28]. Of note, presently it is not possible in these observational studies to clearly define the causal pathway. Although some have argued that the higher levels of CD4+ T cell activation in subjects with lower CD4+ T cell counts reflects a homeostatic response to low CD4+ T cell counts [28], others have argued that higher CD4+ T cell activation is mechanistically involved in CD4+ T cell depletion. Support for this latter perspective is based on several consistent observations, including: (1) measures of T cell activation rather than viral load predict outcome in the natural host of SIV [29], (2) measures of T cell activation in humans predict subsequent disease outcome independent of viral load [8], [9], [30], and (3) generalized T cell activation in the absence of SIV/HIV infection can cause CD4+ T cell but not CD8+ T cell depletion [31], [32]. The striking and consistent relationship between viral load and CD8+ T cell activation observed in our study is consistent with a robust literature suggesting that these two properties are highly associated in untreated and treated disease [7], [28]. Viral replication is almost certainly causally associated with CD8+ T cell activation, as the latter parameter decreases consistently in response to combination therapy [33].

We observed no consistent association between replicative capacity and viremia. This lack of an association may be due to several factors, including the use of an assay that only incorporated parts of the patient-derived viral genome. It is also possible (and indeed we believe likely) that the true replicative capacity of the virus in vivo may not directly predict viremia. Bonhoeffer, Coffin and Nowak, for example, predicted that mutations which reduce the capacity of the virus to infect its target cells would lead to reduction in viremia, reduced CD4+ T cell death, expanded numbers of CD4+ T cells and ultimately an increase in viremia back towards the baseline level (with this increase supported by the increased availability of target cells) [34]. Recently, in collaboration with our group, Vaida and colleagues observed that prospective treatment-mediated reductions in viral fitness resulted had minimal effects on viremia but appeared to preserve CD4+ T cells [3].

The oscillations that we have observed in a range of cell populations are indicative of nonlinear dynamics and interactions between the populations. Oscillations are found commonly in many nonlinear dynamic systems, and the interactions between HIV and the immune system are undoubtedly nonlinear [35], [36]. There have been several studies that have noted this phenomenon. Spouge, Shrager and Dimitrov describe oscillatory behavior of HIV spread in tissue culture systems [37], while theoretical models by Nowak and Bangham [38], Phillips et al [39] and Perelson, Kirschner and De Boer [40] suggest oscillatory behavior may occur *in vivo* as well. To our knowledge, ours is the first study that demonstrates the presence of an oscillatory pattern using observed longitudinal clinical data derived from a study designed in part to address these issues.

A mechanistic model based on our data and prior studies is needed to fully understand the significance of the oscillatory associations between CD4+ T cell counts, viremia, and T cell activation. Although such a model is outside the scope of this paper, it is clear from our current data that, among reasonably stable subjects with drug-resistant HIV, there exists complex cell/virus interaction as indicated by the presence of sine waves at multiple frequencies in the spectral analysis. The presence of two and more frequencies of oscillation is a signature of more than one equilibrium state between the virus and the cells of the immune system. Furthermore, the existence of oscillations of high and low frequencies within the same system is indicative of processes taking place on different time scales. A variety of forces have been identified as drivers of periodicity in natural systems. Dynamic interactions between host and parasite populations cause oscillations in disease epidemics [41]. Climatic determinants have been described as the force behind epidemics of dengue fever and malaria [42], [43], [44]. Random noise is also capable of inducing oscillations in deterministic systems [45]. Such systems are commonly studied using spectral analysis, which is an important tool in describing oscillatory behavior as it provides additional insight into the complexity of system dynamics which could not be gained from analysis solely in the time-domain.

One caveat is that this study is based on a small population of patients who were selected because they had a steady-state viral load on a partially suppressive stable regimen. Further, for the spectral analysis and nonlinear mixed-models patients were selected if they had at least 20 longitudinal observations. It was necessary to have this many time points to be able to identify the sometimes damped oscillatory behavior. Moreover, it is known that co-morbidities such as cardiovascular disease and diabetes can cause generalized immune activation. Although these patients did not have a coronary heart disease or diabetes diagnosis, we did not have data on markers that could indicate a preclinical condition. Further we did not have data on other potential confounders such as exposure to influenza, recent vaccinations or stress that could have caused increases in immune activation. Further detailed studies are needed. Although our study design does not allow for strong conclusions regarding the cause and effect relationship of activation and either viral load or CD4+ T cell counts, it does provide novel insights into this relationship and suggests that it may be altered by drug-resistant HIV. Along these lines, Bonhoeffer and colleagues predicted that a treatment-related decrease in viral fitness would result in a new dynamic between viral load and target cells [27]. Specifically, any decrease in fitness may act to preserve CD4+ T cell counts (due to reduction in pathogenicity); these preserved CD4+ T cells could increase as a consequence, although preservation of total body CD4+ T cells may or may not be reflected in the number of circulating cells. The critical role of target cell availability as a determinant of viral load in treated disease has also been postulated as cause of viral blips during treatment [46], [47].
